# Supplementary material for: Chemoprophylaxis vaccination with a Plasmodium liver stage autophagy mutant affords enhanced and long-lasting protection
Source: NPJ Vaccines. 2021 Aug 10;6:98. doi: 10.1038/s41541-021-00360-1 (PMC8355287; doi:10.1038/s41541-021-00360-1)
Supplement: Supplementary file 1 — Supplementary Information [file 41541_2021_360_MOESM1_ESM.pdf]

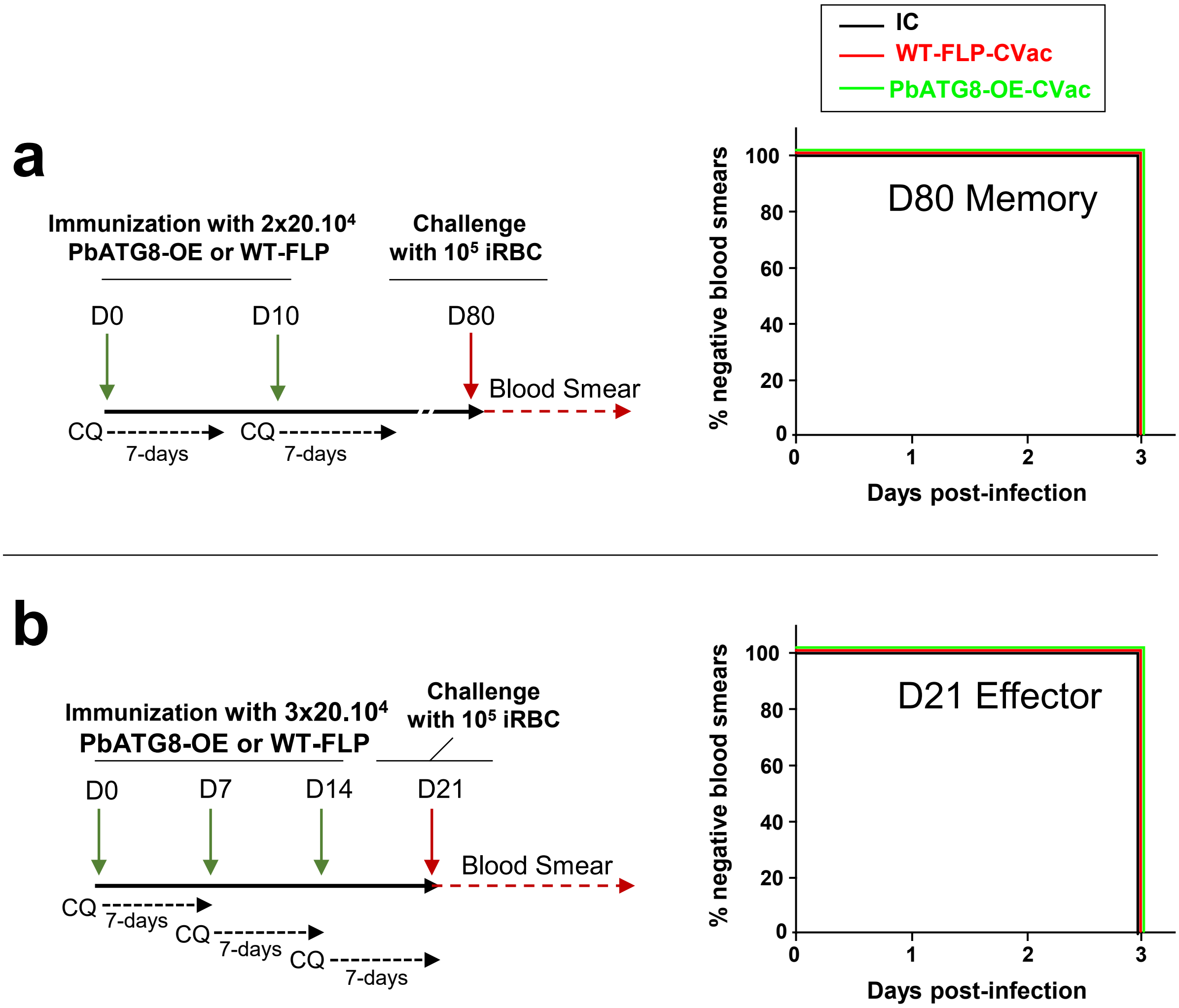

Supplementary Figure 1

PbATG8-OE-CVac-immunized mice are not protected from blood stage challenge

a. Protocol for an immunization with PbATG8-OE-CVac or WT-FLP-CVac and effector challenge with iRBC at D21. Kaplan-Meier survival plots showing days to blood stage infection after challenge. **b.** Protocol for an immunization with PbATG8-OE-CVac or WT-FLP-CVac and challenge with iRBC at D80. For a and b, data are representative of one experiment with 5 mice in each group. IC: non-immunized infection control. No statistical significance determined by log-Mantel Cox test.

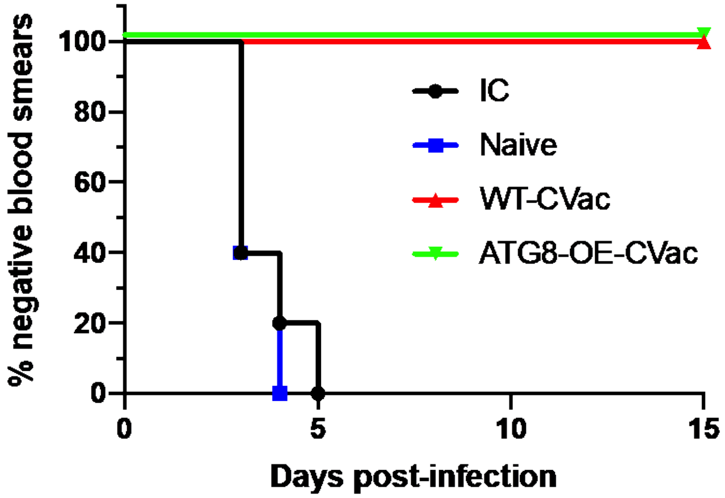

Supplementary Figure 2

Confirmation of SNA in Figure 8b [Antibody response generated by PbATG8-OE-CVac is protective]

SNA to monitor infection in vivo. $2x10^4$ WT-Pb-ANKA sporozoites were incubated on ice for 45 min with 1:6 diluted sera from naïve or immunized mice, and the sporozoite-antibody mixture was injected intradermally to naïve Swiss-Webster mice and parasitemia was monitored for 15 days.
